# PINK1 Alleviates Cognitive Impairments *via* Attenuating Pathological Tau Aggregation in a Mouse Model of Tauopathy

**DOI:** 10.3389/fcell.2021.736267

**Published:** 2022-01-04

**Authors:** Xing Jun Jiang, Yan Qing Wu, Rong Ma, Yan Min Chang, Lu Lu Li, Jia Hui Zhu, Gong Ping Liu, Gang Li

**Affiliations:** ^1^ Department of Neurology, Union Hospital, Tongji Medical College, Huazhong University of Science and Technology, Wuhan, China; ^2^ Department of Pharmacology, School of Basic Medicine, Tongji Medical College, Huazhong University of Science and Technology, Wuhan, China; ^3^ Department of Pathophysiology, School of Basic Medicine and the Collaborative Innovation Center for Brain Science, Key Laboratory of Ministry of Education of China and Hubei Province for Neurological Disorders, Tongji Medical College, Huazhong University of Science and Technology, Wuhan, China; ^4^ Co-Innovation Center of Neuroregeneration, Nantong University, Nantong, China

**Keywords:** tau, PINK1, autophagy, memory, Alzheimer’s disease

## Abstract

As a primary cause of dementia and death in older people, Alzheimer’s disease (AD) has become a common problem and challenge worldwide. Abnormal accumulation of tau proteins in the brain is a hallmark pathology of AD and is closely related to the clinical progression and severity of cognitive deficits. Here, we found that overexpression of phosphatase and tensin homolog (PTEN)-induced kinase 1 (PINK1) effectively promoted the degradation of tau, thereby rescuing neuron loss, synaptic damage, and cognitive impairments in a mouse model of tauopathy with AAV-full-length human Tau (hTau) injected into the hippocampal CA1 area (hTau mice). Overexpression of PINK1 activated autophagy, and chloroquine but not MG132 reversed the PINK1-induced decrease in human Tau levels and cognitive improvement in hTau mice. Furthermore, PINK1 also ameliorated mitochondrial dysfunction induced by hTau. Taken together, our data revealed that PINK1 overexpression promoted degradation of abnormal accumulated tau *via* the autophagy–lysosome pathway, indicating that PINK1 may be a potential target for AD treatment.

## Introduction

Tauopathies are a group of human neurological disorders, which are pathologically characterized by abnormal accumulation of tau filaments in the brain. Among the tauopathies, Alzheimer’s disease (AD) is the most studied (Tapia-Rojas et al., 2019). The main hallmarks of AD pathology are intracellular deposition of tau neurofibrillary tangles and extracellular amyloid-β (Aβ) plaques. Despite being initially considered as a pathological change driven by the toxic effects of amyloid peptide, our understanding of the role that tau plays in AD has been continuously evolving (Götz et al., 2019). Growing evidence indicates that tau pathology can also exert synergistic effects with amyloid peptide and that it correlates more closely to the progression and cognitive impairment of AD than Aβ plaques (Bejanin et al., 2017; Guo et al., 2020). Additionally, given the failure of various clinical Aβ-directed therapies, more efforts have been focused on exploring tau-targeted therapies worldwide in recent years (Congdon and Sigurdsson, 2018).

Phosphatase and tensin homolog (PTEN)-induced kinase 1 (PINK1), a serine/threonine kinase mainly localized to mitochondria, has attracted more and more attention since its mutation was identified in hereditary early-onset Parkinson’s disease (PD) (Valente et al., 2004; Rasool and Trempe, 2018). PINK1 is widely distributed across multiple tissues and organs of the human body, with the brain being the region with the highest expression (Fagerberg et al., 2014). Under normal conditions, after being imported to the mitochondrial membrane, PINK1 is cleaved by mitochondrial presenilin-associated rhomboid-like (PARL) protease, after which it is transported into the cytoplasm, where it gets degraded. Upon mitochondrial depolarization, PINK1 is stabilized and activated on the mitochondrial membrane, and can initiate PINK1-Parkin dependent mitophagy (Arena and Valente, 2017). Beyond its originally perceived role as an initiator of mitophagy, PINK1 has also shown to be involved in regulating autophagy–lysosome pathway (ALP), ubiquitin–proteasome system (UPS), neurite outgrowth and neuron survival, inflammation, tumor suppression, and apoptosis (Michiorri et al., 2010; McLelland et al., 2014; Parganlija et al., 2014; Akabane et al., 2016; Arena and Valente, 2017; Sliter et al., 2018). In addition to PD, PINK1 has also been proven to exert neuroprotective effects in other neurodegenerative diseases, including Huntington’s disease, amyotrophic lateral sclerosis, and AD (Khalil et al., 2015; Quinn et al., 2020; Baek et al., 2021).

Several studies have reported abnormal expression of PINK1 in patients with AD as well as in cellular and animal models of AD. Among them, most studies showed decreased levels of PINK1 in the context of AD pathology (Choi et al., 2014; Du et al., 2017; Manczak et al., 2018; Reddy et al., 2018; Fang et al., 2019; Ochi et al., 2020; Zhao et al., 2020; Liang et al., 2021), although there are also a minority of studies with opposing conclusions (Mise et al., 2017; Pakpian et al., 2020; Zheng et al., 2020). Researchers showed that the increasing expression of PINK1 lessened Aβ plaques accumulation and rescued cognitive impairments in AD mice. The underlying mechanisms probably included induction of the autophagy pathway, altering APP transcription or secretases, increasing the phagocytosis of Aβ plaques by microglia, promoting mitophagy, and improving mitochondrial function (Du et al., 2017; Fang et al., 2019; Han et al., 2020). However, the specific impact of PINK1 on tau pathology remains largely underexplored and existing relevant and targeted studies are suggestive but inadequate. A previous study showed that activation of mitophagy reduced tau levels, while PINK1 knockdown abolished this effect (Fang et al., 2019). In a different study, G309D PINK1 mutation led to a significant increase in phosphorylated tau (Ser396/404) through inhibition of GSK3β activation in cells (Ye et al., 2015). More efforts are needed to establish the precise role of PINK1 in tau pathology and its possible underlying mechanisms.

Here, using a mouse model of tauopathy injected with AAV2-full-length human TAU into the hippocampus, we showed that upregulation of PINK1 significantly alleviated the deposition of pathological tau, neuron loss, synaptic damage, and cognitive impairments in mice. This occurred through inducing tau degradation *via* ALP, reducing tau accumulation in mitochondria and ameliorating mitochondrial disorders. Taken together, our study supports that PINK1 may be a promising target for AD treatment.

## Materials and Methods

### Animals

Wild-type C57BL/6J mice (male, 8–10 weeks-age, 20–25 g) were acquired from Beijing Vital River Laboratory Animal Technology Co., Ltd. Animals were randomly assigned into cages (4–5 mice per cage), under normative cultured environment: 12-h day–night cycle with freely available food and water. All animal experiments were performed according to the “Policies on the Use of Animals and Humans in Neuroscience Research” revised and approved by the Society for Neuroscience in 1995, the Guidelines for the Care and Use of Laboratory Animals of the Ministry of Science and Technology of the People’s Republic of China, and the Institutional Animal Care and Use Committee at Tongji Medical College. The animal study was reviewed and approved by Ethics Committee of Tongji Medical College, Huazhong University of Science and Technology.

### Stereotactic Brain Injection and Drug Administration

pAAV-SYN-human Tau-mCherry-3×FLAG-WPRE (1.30 × 10^13^ vg/ml), pAAV-SYN-PINK1-EGFP-3×FLAG-WPRE (1.35 × 10^13^ vg/ml), and corresponding vehicles pAAV-SYN-MCS-mCherry-3×FLAG (2.09 × 10^13^ vg/ml) and pAAV-SYN-MCS-EGFP-3×FLAG (2.84 × 10^13^ vg/ml) were generated by OBio Tech. Inc. (Shanghai, China). After being fixed on stereotaxic apparatus with adequate anesthesia, mice were injected with 1 µl of virus into the hippocampal CA1 area bilaterally (AP-1.94, ML ± 1.2, DV-1.6). Injection rate was maintained at 100 nl/min, and the needle syringe was kept *in situ* for an additional 10 min after the virus was fully injected. Before putting them back into cages, mice were placed on an electric blanket for revival.

Fourteen days after virus injection, mice were treated with (1) the autophagy inhibitor chloroquine (CQ) (C6628, Sigma-Aldrich) at 50 mg/kg body weight (Campos et al., 2020; Chen et al., 2020), (2) the proteasome inhibitor MG132 (M8699, Sigma-Aldrich) at 0.5 mg/kg body weight (Lu et al., 2017), or (3) the same volume of vehicle daily for 16 days *via* intraperitoneal injection.

### Behavior Tests

One month after the stereotactic injection, behavioral experiments were conducted to evaluate the spatial learning and memory capabilities of the mice. The novel object recognition (NOR) test is a learning and memory evaluation method based on the principle that animals are born with a tendency to explore new things. This test was conducted as follows (Hong et al., 2020): 24 h before the test, mice were placed in the arenas (50 cm × 50 cm container) without objects for a 5-min habituation. The next day, mice were put into the arenas (one sidewall with two identical objects A and A′ separately located on either end) for 5 min. One hour later, object A′ was replaced by a different object, object B, and then the animals were put into the arenas again and allowed to explore both objects for 5 min. A video camera above the arenas logged the experimental behavior. The time that mice spent exploring object A and object B was recorded as TA and TB, respectively. TB/(TA + TB) was set as the recognition index.

The Morris water maze (MWM) test is used to assess the learning and memory abilities of laboratory animals in the context of spatial position and orientation (Morris, 1984). It was performed as follows: during the spatial learning phase, mice were trained to find a concealed platform in a fixed position below the waterline for five consecutive days. The training time was fixed at 12:00 pm-17:00 pm. In each training session, mice were gently put into water from one of the other three quadrants (without target platform), facing the pool wall. If the platform was not sought out within 60 s, mice would be directed to the platform and made to stay in it for another 30 s. On day 7, the platform was removed, and mice were put in the water maze for 60 s to test spatial memory. The motion trails of mice were recorded and analyzed using MWZ-100 system (Techman, China).

### Protein Extraction

Hippocampal regions infected with virus were isolated and mechanically homogenized in lysis buffer for Western blotting (P0013, Beyotime). Homogenate was mixed with 8% (wt/vol) SDS buffer and boiled for 10 min. The sample was further disintegrated through sonication and centrifugation at 12,000 ×*g* for 15 min at 4°C. Supernatant was collected as total protein extract.

For preparation of sarkosyl soluble/insoluble fractions (Goedert et al., 1992; Schlegel et al., 2019; Ferrer et al., 2020), the sample was mechanically homogenized in 10 volumes (w/v) of pre-cooling lysis buffer (10 mM Tris-HCl, pH 7.4, 0.8 M NaCl, 1 mM EGTA, 10% sucrose) and then centrifuged at 20,000 ×*g* for 20 min at 4°C. The supernatant (S1) was transferred to a new Eppendorf tube, and the pellet was re-homogenized in 5 volumes (w/v) of lysis buffer and spun at 20,000 ×*g* for 20 min. The supernatant (S2) was mixed with supernatant (S1) and incubated with 1% N-lauroylsarkosynate (w/v) for 1 h at room temperature while shaken. The sample was then spun at 100,000 ×*g* for 1 h at 4°C. The supernatant was transferred to a new Eppendorf tube, designated as the soluble fraction. The pellet was re-suspended (0.2 ml/g) in 50 mM Tris–HCl (pH 7.4) and stored as sarkosyl insoluble fraction.

To separate out mitochondria from cytoplasm, we used the Tissue Mitochondria Isolation Kit (C3606, Beyotime). Following manufacturer’s instructions, tissue sample was homogenized in solution A and centrifuged at 1,000 ×*g* for 10 min at 4°C. Supernatant was collected and centrifuged at 11,000 ×*g* for another 10 min at 4°C. Then, supernatant was transferred to a new Eppendorf tube, designated as cytoplasm fraction. The deposit was re-suspended in a lysis buffer supplied by the kit and stored as mitochondrial fraction.

### Co-Immunoprecipitation

Hippocampal regions infected with virus were isolated and mechanically homogenized in lysis buffer for immunoprecipitation (IP) (P0013, Beyotime). Then, the homogenate was centrifuged at 3,000 rpm, for 20 min at 4°C. The supernatant was incubated with the primary antibody Tau5 (2 µg/100 µg) (ab80579, Abcam) overnight at 4°C and then Protein A+G Agarose (30 µl/100 µl) (P2012, Beyotime) was added into the sample for 4–6 h. After that, the agarose was washed three times. Proteins attached to the agarose were resuspended in buffer (50 mM Tris-HCl, pH 6.8, 2% SDS, 10% glycerol) and boiled for 10 min. Collected protein sample was analyzed by Western blot.

### Western Blot

*Via* SDS acrylamide gel electrophoresis, protein sample was transferred to nitrocellulose filter membrane (10600002, Whatman) and then blocked in 5% skimmed milk for 1 h. The membrane was then incubated with primary antibodies overnight at 4°C and then incubated with secondary antibody for 1 h. Antibodies used in this study are listed in Table 1. Odyssey Infrared Imaging System (LI-COR Biosciences, Lincoln, NE, United States) and ECL Imaging System (610007-8Q, Clinx Science Instruments Co., Ltd.) were used for visualization of protein bands. Quantitative analysis of blots was performed using ImageJ software (Fiji) (Li et al., 2021).

**TABLE 1:** Antibodies used in this study.

Antibody	Host	DilutionWB	DilutionIHC	DilutionIF	Source
Anti-PINK1	Rabbit	1:500			BC100-494, Novus Biologicals
Anti-PINK1	Rabbit	1:500			ab23707, Abcam
HT7	Mouse	1:1,000	1:100		MN1000, Thermo Fisher Scientific
Tau5	Mouse	1:1,000			ab80579, Abcam
Anti-Tau (pS396)	Rabbit	1:1,000	1:100		11102, Signalway Antibody
Anti-Tau (pS404)	Rabbit	1:1,000	1:100		11112, Signalway Antibody
Anti-Tau (pT205)	Rabbit	1:1,000			11108, Signalway Antibody
Anti-GAPDH	Mouse	1:5,000			60004-1-Ig, Proteintech
Anti-LC3B	Rabbit	1:1,000			ab51520, Abcam
Anti-P62/SQSTM1	Rabbit	1:1,000			18420-1-AP, Proteintech
Anti-LAMP2	Mouse	1:1,000			66301-1-Ig, Proteintech
Anti-Beclin1	Rabbit	1:1,000			11306-1-AP, Proteintech
Anti-Ubiquitin	Mouse	1:1,000			sc-8017, Santa Cruz Biotechnology
Anti-Parkin	Mouse	1:1,000			4211S, Cell Signaling Technology
Anti-COX IV	Rabbit	1:1,000			11242-1-AP, Proteintech
Anti-Caspase 3	Rabbit	1:1,000			9662S, Cell Signaling Technology
Anti-Cleaved caspase 3	Rabbit	1:500			9661S, Cell Signaling Technology
Anti-EGFP	Rabbit			1:100	GB11602, Servicebio
Anti-Iba1	Mouse			1:100	GB12105, Servicebio
Anti-mouse IgG	Goat	1:10,000			A23910, Abbkine
Anti-rabbit IgG	Goat	1:10,000			A23920, Abbkine
Anti-mouse IgG	Goat	1:3,000			A25012, Abbkine
Anti-mouse IgG	Goat	1:5,000			SA00001-1, Proteintech
Anti-rabbit IgG	Goat	1:5,000			SA00001-2, Proteintech
Anti-NeuN	Rabbit		1:200		ab177487, Abcam
Anti-NeuN	Mouse			1:200	ab104224, Abcam
Anti-mouse IgG	Goat		1:200		G1216-3, Servicebio
Anti-rabbit IgG	Goat		1:200		G1215-3, Servicebio
Anti-rabbit IgG	Donkey			1:200	ANT024S, Antgene
Anti-mouse IgG	Donkey			1:200	ANT029S, Antgene

WB, Western blot; IHC, immunohistochemical staining; IF, immunofluorescence staining.

### Quantitative Real-Time PCR

Total RNA was isolated from virus-infected mice hippocampal region using Trizol reagent (15596018, Thermo Fisher Scientific). The transcription reagent kit (RR037, Takara) was then used for cDNA synthesis. Quantitative PCR was conducted using the One-Step SYBR PrimeScript PLUS RT-PCR Kit (RR096A, Takara) following the manufacturer’s instructions. The PCR system contained 1 µl of forward and reverse primers, 1 µl of cDNA, 3 µl of diethylpyrocarbonate (DEPC H_2_O), and 5 µl of SYBR Green PCR master mixes. RT-PCR was performed and analyzed using an ABI Step one plus Real-Time PCR System (Applied Biosystems). Primers for human *TAU* were F: 5′-CGC​CAG​GAG​TTC​GAA​GTG​AT-3′ and R: 5′-TCT​TGG​TGC​ATG​GTG​TAG​CC-3′ (Korhonen et al., 2011) and primers for *β-actin* were F: 5′- CAA​ATG​TTG​CTT​GTC​TGG​TG-3′ and R: 5′-GTC​AGT​CGA​GTG​CAC​AGT​TT-3′ (Wan et al., 2021).

### Immunohistochemical and Nissl’s Staining

Mice brain slices (paraffin section, 4 µm thick) were baked at 55°C for 1 h and immersed into xylene for 40 min. After being dewaxed, slices were dehydrated through graded ethanol (100%, 100%, 95%, 90%, and 80%) for 5 min each time. Brain slices were immersed into citric acid buffer (pH = 6.0, 10 mM) and heated in a microwave for 10 min to maximize tissue antigen recovery. Then, slices were incubated with 3% H_2_O_2_ for 30 min and blocked in 5% BSA solution containing 0.5% Triton X-100 for 40 min. Next, slices were incubated with primary antibody (listed in Table 1) at 4°C for 24–48 h. After incubation with secondary antibody at 37°C for 1 h, slices were stained with DAB reagent (G1212, Servicebio). Then, slices were rehydrated through graded ethanol (80%, 90%, 95%, 100%, and 100%) for 5 min each time, transparentized using xylene for 20 min, and mounted with neutral balsam. For Nissl’s staining, after deparaffinage and gradient alcohol dehydration, slices were washed with PBS for 3 × 5 min, and then dyed with 0.5% toluidine blue reagent (G1036, Servicebio) for 2–5 min. If the slice was hyperchromatic, 0.1% glacial acetic acid was used for differentiation. Baked slices were mounted with neutral balsam. A scanning microscope (SV120, OLYMPUS) was used for imaging.

### Immunofluorescence Staining

After aforementioned deparaffinage, dehydration, and antigen recovery, mice brain slices were washed with PBS for 3 × 5 min and blocked in 5% donkey serum containing 0.5% Triton X-100 for 40 min. Next, slices were incubated with primary antibody (listed in Table 1) at 4°C for 24–48 h. After incubation with secondary antibody at 37°C for 1 h, slices were washed with PBS for 3 × 5 min and stained with DAPI reagent (G1012, Servicebio) for 10 min at room temperature. Slices were sealed with anti-fluorescence quencher (G1401, Servicebio) and imaged using a scanning microscope (SV120, OLYMPUS).

### Golgi Staining

FD Rapid GolgiStain^™^ Kit (FD Neuro Technologies, PK401) was used for Golgi staining. After being deeply anesthetized, the brain of mice was removed and immersed in mixture solution A + B (1:1) for 2–4 weeks. Then, the brain was transferred to solution C for 3–7 days, after which it was placed on an oscillating tissue slicer and cut into slices (100 µm thick). After being air dried in the dark, the slices were stained with a mixture of solution D + E + double distilled water (1:1:2) as per manufacturer’s instructions. Images were taken using an optical microscope (Nikon, Japan).

### ATP Assay

ATP levels were measured using the ATP bioluminescence detection kit (S0026, Beyotime). Briefly, the hippocampal regions infected with virus was extracted and pyrolyzed with a lysis buffer supplied with the kit. The homogenate was centrifuged at 12,000 ×*g* for 5 min at 4°C. Supernatant was collected for ATP detection. Protein concentration of the supernatant was measured using the BCA Protein Assay Kit (P0012S, Beyotime). Furthermore, 100 µl of supernatant and 100 µl of ATP detection buffer were mixed and the luminescence was measured using a microplate reader. Gradient dilution of the standard solution was conducted to generate the standard curve (1 nM–1 µM). ATP levels were calculated according to the standard curve and normalized against the standards’ protein concentration.

### Malondialdehyde Assay

Malondialdehyde (MDA) levels were measured using the Lipid Peroxidation MDA Assay Kit (S0131S, Beyotime). As per manufacturer’s instructions, hippocampal regions infected with virus was extracted and pyrolyzed in RIPA lysis buffer (P0013D, Beyotime) and then centrifuged at 12,000 ×*g* for 10 min at 4°C. Supernatant was collected for MDA detection. Protein concentration of the supernatant was measured using a BCA Protein Assay Kit (P0012S, Beyotime). One hundred microliters of supernatant was mixed with 200 µl of MDA detection working buffer containing thiobarbituric acid (TBA) and the MDA-TBA adduct was measured using a microplate reader at 535 nm. Gradient dilution of standard solutions was conducted to generate the standard curve (1–100 µM). MDA levels were calculated according to the standard curve and normalized against the standards’ protein concentration, shown as nmol/mg protein.

### Statistical Analysis

All data were collected and analyzed in a blinded manner. Data were shown as mean ± SEM or mean ± SD and analyzed using GraphPad Prism (GraphPad Software, Inc., La Jolla, CA, United States). Statistical analyses were conducted using two-tailed unpaired *t*-tests, one-way ANOVA, or two-way repeated measures ANOVA followed by Tukey multiple-comparisons post-hoc tests. *p* < 0.05 was set as the level of statistical significance.

## Results

### PINK1 Rescues Cognitive Impairments in hTau Mice

**FIGURE 1:**
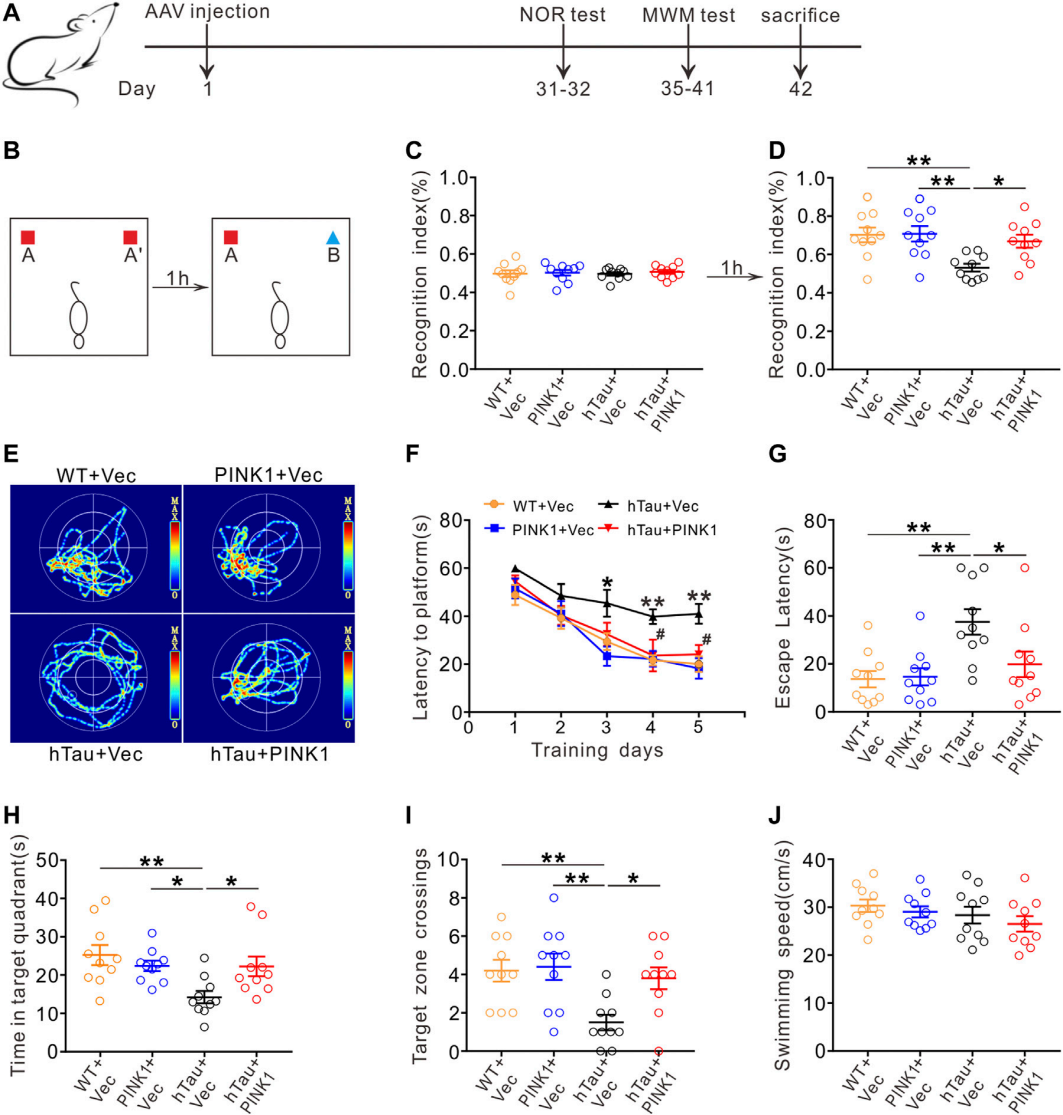
PINK1 ameliorates cognitive impairments in hTau mice. **(A)** Experimental processes of virus injection and behavioral tests. **(B–D)** PINK1 improved cognitive performance of hTau mice in the NOR test shown by elevated recognition index. One-way ANOVA followed by Tukey multiple-comparisons tests. *p < 0.05, **p < 0.01. **(E)** Representative swimming path of mice in each group during the MWM probe test. **(F)** PINK1 improved learning ability in hTau mice shown by shortened latency to find the hidden platform during training stage in the MMW test. Two-way repeated-measures ANOVA followed by Tukey multiple-comparisons tests. *p < 0.05, **p < 0.01 vs. WT + Vec; ^#^ *p* < 0.05 vs. hTau + Vec. **(G–I)** PINK1 improved memory ability in hTau mice shown by decreased latency to reach the location of platform **(G)**, longer retention time in the target quadrant **(H)**, and more target zone crossings **(I)** during the MWM probe test. One-way ANOVA followed by Tukey multiple-comparisons tests. *p < 0.05, **p < 0.01. **(J)** No significant difference in swimming speed was seen among the four groups during the MWM probe test. One-way ANOVA followed by Tukey multiple-comparisons tests. All data were presented as mean ± SEM. *n* = 10 mice for each group.

We injected pAAV-SYN-human Tau-mCherry-3×FLAG-WPRE into the hippocampal CA1 region of mice for 1 month to mimic Alzheimer-like deposits of tau in the brain (Andorfer et al., 2003; Lasagna-Reeves et al., 2011; Li et al., 2019; Wan et al., 2021). Meanwhile, pAAV-SYN-PINK1-EGFP-3×FLAG-WPRE was also co-injected to explore its effect on tau pathology. SYN is a neuron-specific promoter, which means exogenous PINK1 and hTau would be specifically expressed in neurons. A high transfection efficiency of the virus was confirmed by Western blot, immunofluorescence and immunohistochemistry (Figures 4A,B,E; Supplementary Figure S1). As core symptoms of AD, cognitive decline and dementia are tightly associated with tau pathology (Bejanin et al., 2017). Thus, we conducted behavioral tests on the mice to assess cognitive function (Figure 1A). In contrast with WT mice, human Tau (hTau) mice (injected with pAAV-SYN-human TAU-mCherry-3×FLAG-WPRE) showed obvious learning and memory impairments as evaluated by NOR and MWM tests, while overexpression of PINK1 rescued the cognitive dysfunction. More specifically, in the NOR test, the time that hTau mice spent on exploring novel object was significantly reduced; however, this recognition index was improved by PINK1 overexpression (Figures 1B–D). In the MWM test, compared with the WT group, hTau mice had a longer latency period before finding the hidden platform at 3rd–5th days during the training stage. During the test stage, longer latency to reach the place where the platform was previously placed before, less retention time in the target quadrant, and fewer times crossing the platform region were observed in hTau mice, while overexpressing PINK1 attenuated the above learning and memory deficits, as evidenced by decreased time to find the platform during the 4th and 5th day during the training phase, less escape latency, more retention time in the target quadrant, and more times crossing the platform region during the test phase (Figures 1E–I). There was no significant difference in swimming speed among the four groups of mice (Figure 1J), which excluded defects in motor ability. Overall, our data demonstrated that PINK1 overexpression ameliorates cognitive deficits in hTau mice.

### PINK1 ameliorates hTau-Induced Neuron Loss and Synaptic Damage

**FIGURE 2:**
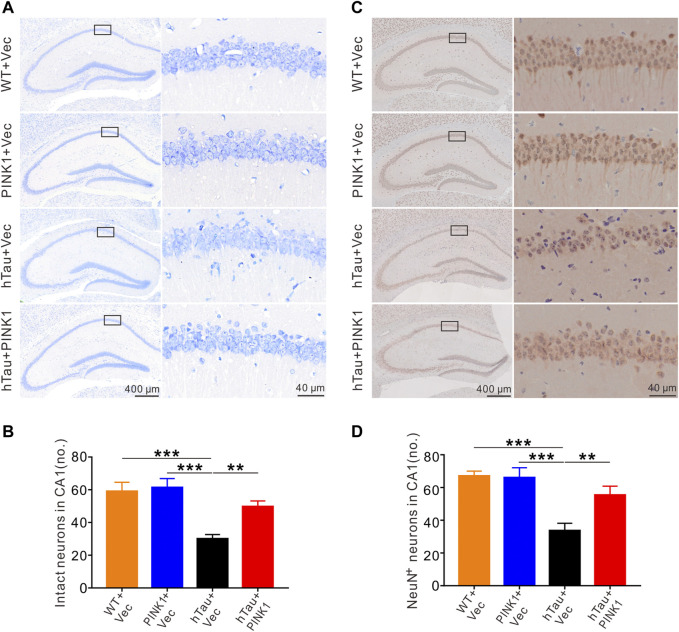
PINK1 alleviates hTau-induced neuronal loss in the hippocampal CA1 region of mice. (**A)** PINK1 ameliorated hippocampal CA1 neuronal loss in hTau mice exhibited by representative images of Nissl staining. **(B)** Quantitative analysis of numbers of intact neurons in area framed within black bordered rectangle. Neurons with visible nuclei, distinctive nucleolus, and cytoplasmic Nissl staining were regarded as intact neurons and counted. One-way ANOVA followed by Tukey multiple-comparisons tests. **p < 0.01, ***p < 0.001. **(C)** Representative images of NeuN immunohistochemical staining. **(D)** Quantitative analysis of numbers of neurons with positive NeuN staining in area framed within black bordered rectangle. One-way ANOVA followed by Tukey multiple-comparisons tests. **p < 0.01, ***p < 0.001. All data were presented as mean ± SD. *n* = 3 mice for each group.

Growing evidence supports the neurotoxic effects of tau as a primary event for neuron loss and synaptic injury, both of which are common neuropathologic manifestations in AD and closely related to the severity of cognitive disfunction (Iqbal and Grundke-Iqbal, 2002; Giannakopoulos et al., 2003). Especially, neuron loss in the hippocampal CA1 region is a prominent feature of AD (West et al., 1994; Simic et al., 1997). Therefore, we aimed to investigate the underlying mechanisms by which PINK1 ameliorated cognitive deficits in hTau mice. Nissl’s staining and immunohistochemical staining were used to observe and compare neuron morphology and number in the hippocampal CA1 area of mice. In WT mice, irrespective of whether injected with PINK1 or not, neurons in the CA1 region had a full and orderly shape and were closely arranged with the Nissl bodies. Meanwhile, hTau mice displayed a reduced number of intact neurons (Figures 2A,B). Neurons had abnormal morphology, with an obscure structure and disorganized arrangement (Figure 2A). Interestingly, this phenotype was noticeably attenuated following PINK1 overexpression (Figures 2A,B). NeuN staining data also supported the finding that PINK1 overexpression ameliorated the neuron loss induced by accumulation of hTau (Figures 2C,D). In addition, overexpression of PINK1 attenuated the observed increased levels of cleaved caspase-3 induced by hTau, thus suggesting that PINK1 alleviates hTau-induced cell apoptosis (Supplementary Figure S2).

**FIGURE 3:**
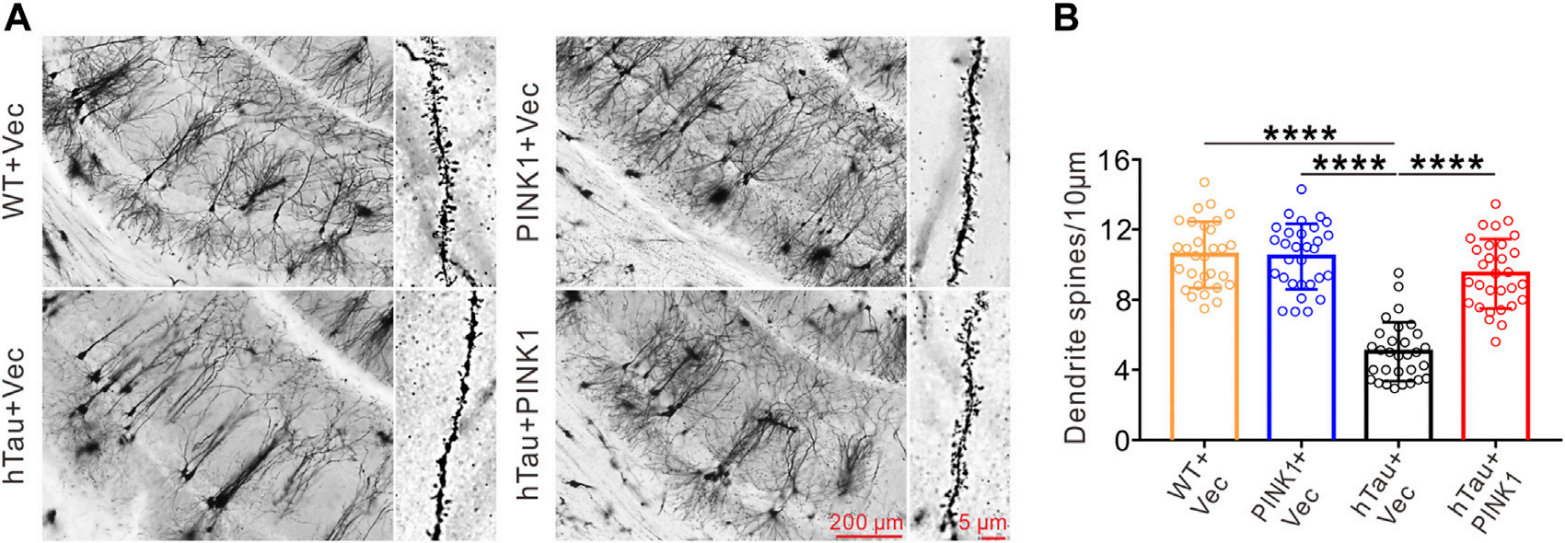
PINK1 reverses the decreased dendritic spine density in the hippocampal CA1 region of hTau mice. **(A)** Representative images of Golgi Staining in the hippocampal CA1 region of mice. **(B)** Quantitative analysis of spine density in the CA1 area of mice. Thirty neurons from each group were analyzed. One-way ANOVA followed by Tukey multiple-comparisons tests. ****p < 0.0001. All data were presented as mean ± SD. *n* = 3 mice for each group.

The dendritic spine, functional protrusions on dendrite branches, is the main site of synaptogenesis, and so is closely related to synaptic transmission (Chidambaram et al., 2019). Here, we identified and quantified dendritic spines in the CA1 region using Golgi staining. Compared with WT mice, dendritic branches were sparser, and the dendritic spine density was significantly reduced in hTau mice (Figure 3). Meanwhile, overexpression of PINK1 attenuated such phenotype in hTau mice (Figure 3). These data indicated that PINK1 attenuates hTau-mediated neuron loss and synaptic damage.

### PINK1 Overexpression Reduces Tau Protein Levels in hTau Mice

**FIGURE 4:**
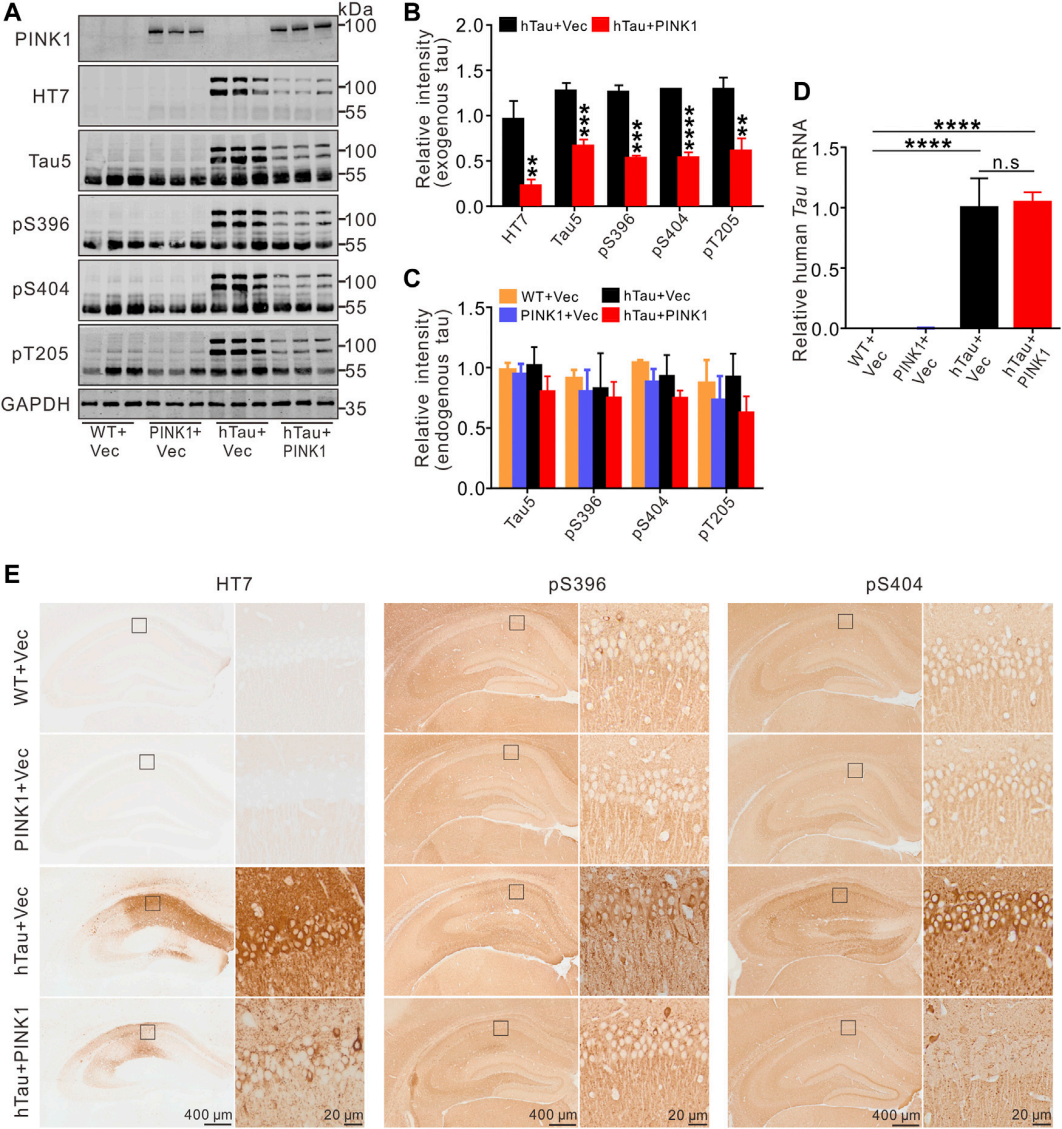
Overexpression of PINK1 decreases tau protein levels in hTau mice. **(A,B)** Representative images and quantitative analysis of Western blot showed overexpression of PINK1 diminished levels of exogenous tau (∼106 kDa, human tau), including total tau (HT7, Tau5) and phosphorylated tau (pS396, pS404, and pT205) in the hippocampal CA1 area of hTau mice. Unpaired *t*-tests. **p < 0.01, ***p < 0.001, ****p < 0.0001. **(C)** Significant alteration of endogenous tau (∼55 kDa) was not found among the four groups. One-way ANOVA followed by Tukey multiple-comparisons tests. **(D)** The levels of human *TAU* mRNA had no significant changes with PINK1 overexpression. One-way ANOVA followed by Tukey multiple-comparisons tests. ****p < 0.0001. **(E)** Representative images of immunohistochemical staining showed PINK1 reduced tau pathology (total tau, phosphorylated tau at Ser396 or Ser404) in the hippocampal CA1 area. HT7 antibody exclusively reacts to human tau proteins. All data were presented as mean ± SD. *n* = 3 mice for each group.

**FIGURE 5:**
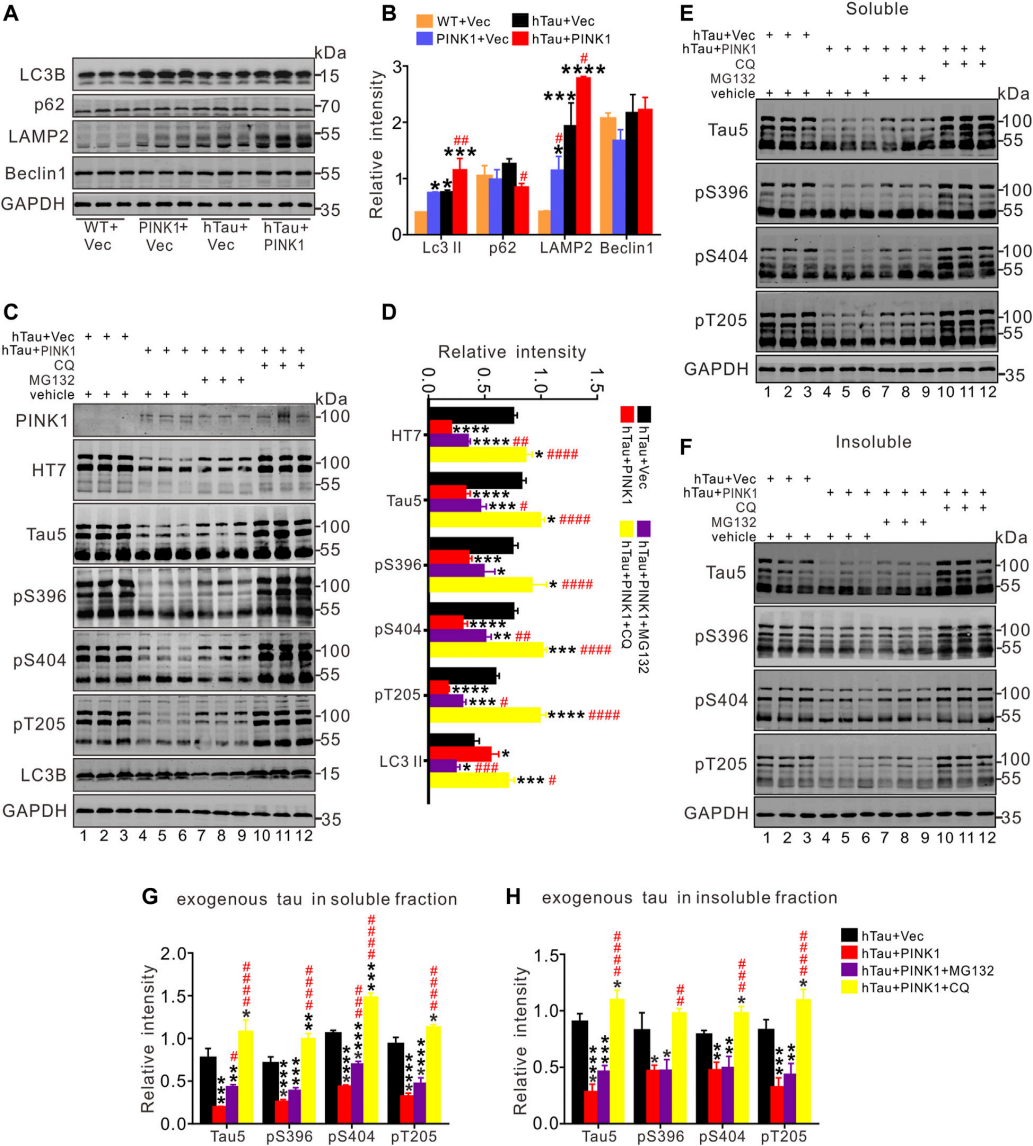
PINK1 promotes clearance of hTau mainly through the autophagy pathway. **(A,B)** PINK1 increased the levels of LC3 II and lysosomal protein *LAMP2*, as well as decreased p62 levels detected by Western blot. One-way ANOVA followed by Tukey multiple-comparisons tests. *p < 0.05, **p < 0.01, ***p < 0.001, ****p < 0.0001 vs. WT + Vec; ^#^ *p* < 0.05, ^##^ *p* < 0.01 vs. hTau + Vec. **(C,D)** In total fraction of mice hippocampal CA1, PINK1 diminished the levels of exogenous tau (∼106 kDa, human tau: both total and phosphorylated tau), while CQ treatment reversed this reduction shown by Western blot. Treatment with MG132 induced relatively small increase in the levels of hTau proteins in hTau and PINK1 overexpressing mice. **(E–H)** In both the soluble **(E, G)** and insoluble fraction **(F, H)** of mice hippocampal CA1 area, PINK1 decreased the levels of exogenous tau (∼106 kDa, human tau: both total and phosphorylated tau) in hTau mice, while CQ treatment reversed the reduction. Treatment with MG132 induced relatively small increase in the levels of hTau proteins in the soluble fraction. One-way ANOVA followed by Tukey multiple-comparisons tests. *p < 0.05, **p < 0.01, ***p < 0.001, ****p < 0.0001 vs. hTau + Vec; ^#^ *p* < 0.05, ^##^ *p* < 0.01, ^###^ *p* < 0.001, ^####^ *p* < 0.0001 vs. hTau + PINK1. All data were presented as mean ± SD. *n* = 3 mice for each group.

We observed obvious accumulation of exogenous tau (human Tau) proteins in the hippocampal CA1 region of hTau mice, including total human tau (detected using the HT7 and Tau5 antibodies) and phosphorylated tau at Ser396, Ser404, and Thr205 (Figures 4A,B,E), as detected by Western blot or immunohistochemistry. Simultaneously overexpressing PINK1 significantly reduced the levels of exogenous total and phosphorylated tau proteins (Figures 4A,B,E). Furthermore, we found that PINK1 overexpression decreased soluble and insoluble exogenous total and phosphorylated tau proteins compared with hTau mice (Figures 5E–H, 1–3 lane vs. 4–6 lane). PINK1 did not alter the mRNA levels of hTau (Figure 4D), which suggested that overexpressing PINK1 decreased hTau protein levels as a result of an increase in its degradation. Although the endogenous levels of mouse tau displayed a downward trend in the context of PINK1 overexpression, there was no significant difference among the four groups (Figures 4A,C). Overall, these data showed that PINK1 decreases the pathological accumulation of tau proteins induced by hTau overexpression.

### PINK1 Promotes Clearance of hTau Accumulation *via* the Autophagy–Lysosome Pathway

Given the protective effects of PINK1 against abnormally accumulated tau protein in mice, we sought to find out the potential underlying mechanism. As PINK1 did not lead to changes in the mRNA levels of hTau (Figure 4D), we inferred that PINK1 may affect the degradation pathways of hTau proteins. It has been previously shown that both UPS and ALP contribute to the degradation of tau aggregation in AD (Cheng et al., 2018), and there are also data that indicate the promotive effects of PINK1 on ALP (Michiorri et al., 2010; Parganlija et al., 2014; Du et al., 2017). Therefore, we aimed to detect the expression of autophagic markers (Abdelfatah et al., 2021). As illustrated in Figures 5A,B, PINK1 overexpression induced an increase in the expression of LC3 II and lysosomal protein LAMP2, and a decrease in the levels of p62 in the hippocampal CA1 region of hTau mice. The level of Beclin1 was not significantly altered among the four groups. Thus, these results suggest that PINK1 activates ALP in hTau mice.

To further confirm the pathway by which PINK1 induces elimination of abnormal accumulation of tau, MG132, an inhibitor of proteasome pathway, or chloroquine (CQ), an inhibitor of autophagy that blocks the fusion of autophagosome and lysosome, was used to treat the mice overexpressing hTau and PINK1, respectively. We found that CQ (lanes 10–12) but not MG132 treatment (lanes 7–9) reversed the decreased total (Tau5) and phosphorylated tau (pS396, pS404, and pT205) levels induced by PINK1 (Figures 5C,D; Supplementary Figure S3). Soluble and insoluble proteins were extracted, and total or phosphorylated tau levels were detected by Western blot. The levels of total and phosphorylated tau in the soluble and insoluble fraction of hTau mice were decreased following PINK1 overexpression, while CQ reversed the PINK1-induced reduction of total or phosphorylated tau levels (Figures 5E–H, lanes 10–12 vs. lanes 4–6; Supplementary Figure S3). MG132 treatment merely induced a small increase in total and phosphorylated tau levels in the soluble fraction, but had no effects in the levels of total or phosphorylated tau in the insoluble fraction (Figures 5E–H, lanes 7–9 vs. lanes 4–6; Supplementary Figure S3). We also observed that CQ caused an increase in the levels of endogenous tau (Figures 5E,F; Supplementary Figures S4A,B). All these data suggest that PINK1 decreases the levels of tau through the autophagy pathway.

### CQ Reverses the Improved Effects of PINK1 on Cognition

**FIGURE 6:**
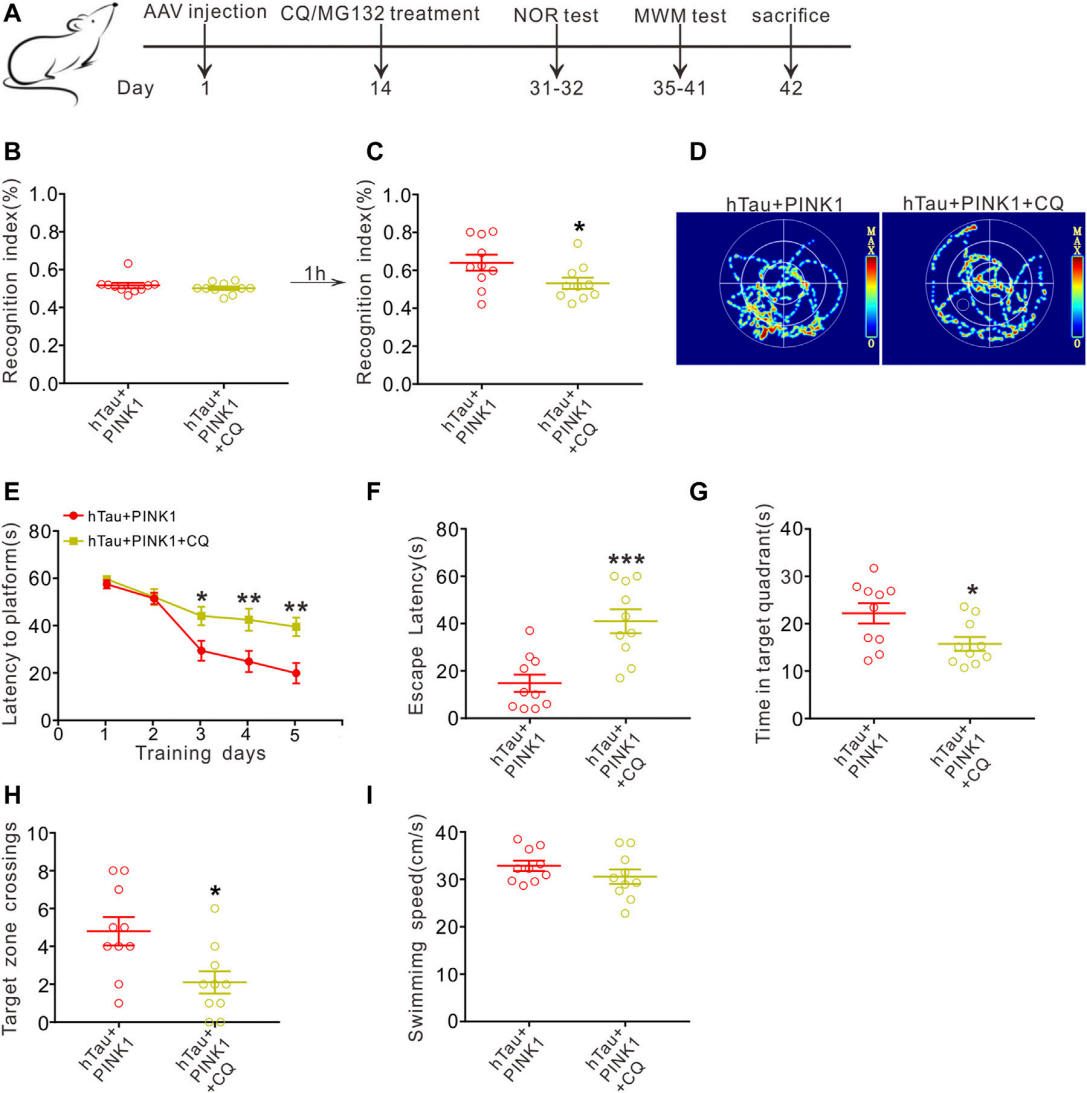
CQ treatment reverses the improved cognition induced by PINK1 overexpression. **(A)** Experimental processes of virus injection, drug treatment, and behavioral tests. **(B,C)** CQ treatment lowered the recognition index of hTau and PINK1 overexpressing mice in the NOR test. Unpaired *t*-tests. *p < 0.05. (D) Representative swimming path of mice in each group during the MWM probe test. **(E)** CQ treatment impaired the learning ability of hTau and PINK1 overexpressing mice, shown by prolonged latency to find the hidden platform during training stage in the MWM test. Two-way repeated-measures ANOVA followed by Tukey multiple-comparisons tests. *p < 0.05, **p < 0.01. **(F–H)** CQ treatment impaired the memory ability of hTau and PINK1 overexpressing mice, shown by longer latency to reach the location of platform **(F)**, shorter retention time in the target quadrant **(G)** and fewer target zone crossings **(H)** during the MWM probe test. Unpaired *t*-tests. *p < 0.05, **p < 0.01, ***p < 0.001. **(I)** No significant difference in swimming speed was seen between the two groups during the MWM probe test. Unpaired *t*-tests. All data were presented as mean ± SEM. *n* = 10 mice for each group.

Furthermore, we conducted behavioral experiments to investigate whether the improved cognitive function induced by PINK1 was also reversed by CQ treatment (Figure 6A). Unsurprisingly, we found that CQ treatment caused a distinct cognitive decline in hTau and PINK1 overexpressing mice. This was evidenced by a lower recognition index in the NOR test (Figures 6B,C), a longer latency period to reach the hidden platform on days 3–5 during the training phase, as well as a longer escape latency, shorter residence time in the target quadrant, and decreased platform zone crossing times during the test phase of the MWM test (Figures 6D–I). Treatment with MG132 did not induce significant changes in the cognition of hTau and PINK1 overexpressing mice (Supplementary Figure S5).

### PINK1 Reduces the Accumulation of hTau in Mitochondria and Improves Mitochondrial Function

**FIGURE 7:**
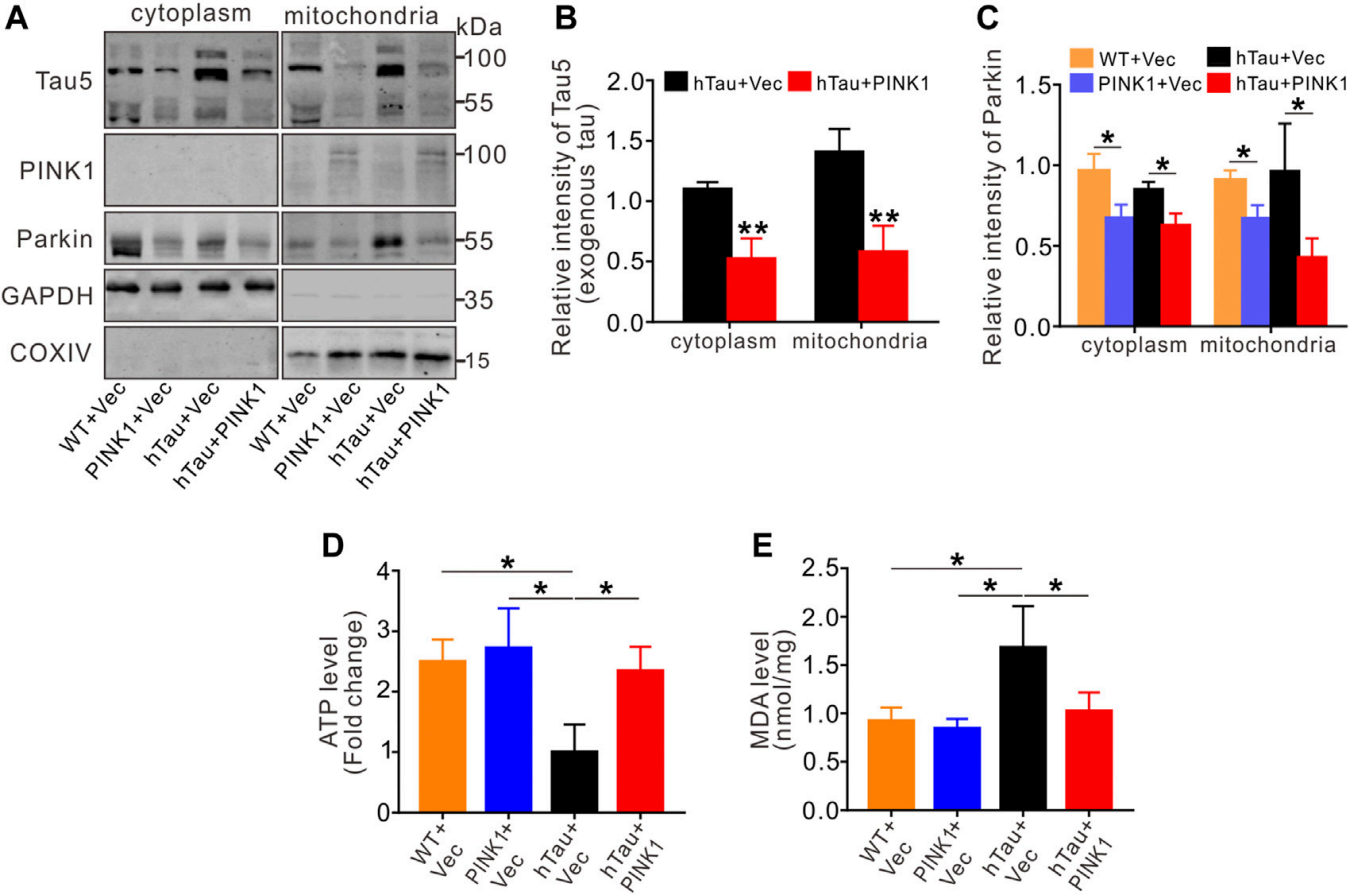
PINK1 reduces tau accumulation in mitochondria and rescues mitochondrial disorders. **(A–C)** The levels of exogenous tau (∼106 kDa, human tau) **(A,B)** or Parkin **(A, C)** in both cytoplasm and mitochondria fraction of mice hippocampal CA1 region were detected by Western blot and quantitative analysis. Unpaired t-tests. *p < 0.05, **p < 0.01. **(D, E)** Overexpression of PINK1 reversed the decreased ATP levels **(D)** or increased MDA levels **(E)** in hTau mice. One-way ANOVA followed by Tukey multiple-comparisons tests. *p < 0.05. All data were presented as mean ± SD. *n* = 3 mice for each group.

Besides our above discovery that PINK1 reduces tau proteins through the autophagy pathway, PINK1 has been widely reported to play vital roles in maintaining mitochondrial homeostasis (Arena and Valente, 2017). Here, we extracted the mitochondrial and cytoplasmic fraction from the hippocampal CA1 region of mice, and found accumulation of hTau in the mitochondrial fraction of hTau mice, which is consistent with our previous study in cells overexpressing hTau (Hu et al., 2016) as well as other previous studies (Lasagna-Reeves et al., 2011; Grassi et al., 2019; Torres et al., 2021), Meanwhile, PINK1 overexpression decreased the levels of hTau in both the mitochondrial and cytoplasmic fraction of hTau mice (Figures 7A,B). Furthermore, we found reduced levels of Parkin in the mitochondrial and cytoplasmic fraction of mice with PINK1 overexpression (Figures 7A,C), indicating an activation of Parkin by PINK1 as activation of Parkin would induce its own ubiquitylation and degradation (Zhang et al., 2000; Xiong et al., 2009; McWilliams et al., 2018). In line with previous studies (Li et al., 2016; Guha et al., 2020; Szabo et al., 2020), assessment of mitochondrial function revealed mitochondrial dysfunction in hTau mice, as evidenced by reduced levels of ATP and elevated MDA levels, both of which were reversed in the context of PINK1 overexpression (Figures 7D,E).

## Discussion

As a primary cause of dementia and death in older people, AD has become a common problem and challenge in an aging society due to a lack of effective diagnosis and treatment. Here, we injected AAV2-full-length human TAU into the hippocampal CA1 region of mice to mimic Alzheimer-like tau pathology in the brain. We observed an obvious accumulation of tau proteins, neuron loss, synapse injury, mitochondrial function disorders, and cognitive impairments in hTau mice. We also found that overexpression of PINK1 effectively reduced neuropathological accumulation of tau proteins, ameliorated mitochondrial function, attenuated damage to neurons and synapses, and thus rescued cognitive decline in hTau mice.

In our study, overexpression of PINK1 led to activation of ALP, as evidenced by increased LC3 II and lysosomal protein LAMP2, as well as decreased levels of p62. PINK1-induced ALP activation has also been observed in other studies (Michiorri et al., 2010; Parganlija et al., 2014; Du et al., 2017). In transgenic mAPP mice, overexpression of PINK1 increased the expression of LC3 II, lysosome-associated membrane protein 1 (LAMP1), lysosomal proteases cathepsin D, autophagy receptor OPTN and NDP52, thus leading to the clearance of Aβ plaques (Du et al., 2017). SH-SY5Y cells with PINK1 knockdown showed decreased mRNA levels of *ATG5*, *ATG6*, *ATG7*, *LC3A*, *P62*, *LAMP1*, and *LAMP2* (Parganlija et al., 2014). PINK1 was also reported to directly interact with Beclin1 to promote autophagy (Michiorri et al., 2010). Taken together, our results indicate that PINK1 promotes degradation of tau *via* ALP. After pharmaceutical blockade of the fusion between autophagosome and lysosome by CQ, we found that the decreased hTau proteins in hTau and PINK1-overexpressed mice, including total and phosphorylated tau in whole tissue homogenate, soluble and insoluble portions, were all reversed. In contrast, inhibiting proteasome by MG132 just induced partial increase of hTau proteins in hTau and PINK1-overexpressed mice. This increase was mainly observed in the soluble portion, which is in accord with the idea that the proteasome has a limited ability to deal with oligomeric and aggregated proteins (Kirkin et al., 2009). CQ also reversed the PINK1-induced improvements on cognitive impairment. Overall, the above findings verified that PINK1 relies on ALP to clear abnormal accumulated hTau proteins and ameliorate cognitive deficits. Thus, in future research, we will explore the detailed mechanism by which PINK1 overexpression leads to the degradation of tau *via* ALP.

Previous research has shown that cytosolic PINK1 fragments enhanced Parkin-mediated ubiquitination and degradation of Parkin substrates in neuroblastoma cells and human brain lysates (Xiong et al., 2009), and bioinformatic analysis presented the potential involvement of Parkin in the ubiquitination of tau (Kumar and Kumar, 2019). Researchers have also shown that Parkin brings about Lys63-linked polyubiquitination of misfolded proteins and leads to their clearance *via* the autophagy pathway (Olzmann and Chin, 2008; Khandelwal et al., 2011). In this study, a Co-IP experiment revealed upregulated ubiquitination of tau protein in the hippocampal CA1 region of mice with PINK1 overexpression (Supplementary Figure S6).

Our previous studies found that hTau can accumulate in mitochondria, inhibit mitophagy, disrupt mitochondrial dynamics, and induce mitochondrial dysfunction in cellular or animal models overexpressing hTau (Hu et al., 2016; Li et al., 2016), which are considered drivers of synaptic dysfunction and cognitive decline in AD (John and Reddy, 2021; Sharma et al., 2021). In this study, we verified the pathological accumulation of tau in mitochondria, which was reported to induce mitochondrial dysfunction, thus contributing to synaptic impairment and memory deficits in mice (Torres et al., 2021). PINK1 has been widely reported to play vital roles in maintaining mitochondrial homeostasis (Voigt et al., 2016; Arena and Valente, 2017). Parkin is a cytosolic member of E3 ubiquitin ligase family, and overexpression PINK1 can recruit Parkin to mitochondria and activate it *via* phosphorylation of its UBL domain. Then, Parkin transfers ubiquitin chains to the mitochondrial outer membrane to induce the elimination of mitochondria through mitophagy (Okatsu et al., 2012; Du et al., 2017; Gundogdu et al., 2021). Our study showed that PINK1 reduced hTau accumulation in mitochondria. We observed mitochondrial dysfunction in hTau mice, which was rescued following overexpression of PINK1, possibly because of PINK1-induced reduction of intracellular tau accumulation (Li et al., 2016; Guha et al., 2020; Szabo et al., 2020) and the direct protective effects of PINK1 on mitochondria (Voigt et al., 2016; Arena and Valente, 2017).

It has been extensively reported that tau accumulation induces neuron loss and synaptic impairments, which are closely related to cognitive deficits in AD (Iqbal and Grundke-Iqbal, 2002; Giannakopoulos et al., 2003; Yin et al., 2016). We also found that overexpression of hTau or P301L hTau activated STAT1 and inactivated STAT3 to inhibit the expression of NMDARs, thus inducing dendritic plasticity deficits, including LTP suppression and spine density decrease, and memory deficits (Li et al., 2019; Hong et al., 2020; Wan et al., 2021). In this study, PINK1 overexpression rescued neuron loss and synaptic damage, and ameliorated cognitive impairments by promoting the degradation of accumulated tau in the autophagy pathway, reducing tau accumulation in mitochondria and alleviating mitochondrial disorders. These findings, together with the previous finding that PINK1 decreased Aβ level in transgenic mAPP mice (Du et al., 2017), indicate the potential of PINK1 as a therapeutic target for AD treatment.

## Data Availability

The original contributions presented in the study are included in the article/Supplementary Material, Further inquiries can be directed to the corresponding authors.
