# Supplementary material for: PINK1 Alleviates Cognitive Impairments via Attenuating Pathological Tau Aggregation in a Mouse Model of Tauopathy
Source: Front Cell Dev Biol. 2022 Jan 4;9:736267. doi: 10.3389/fcell.2021.736267 (PMC8763800; doi:10.3389/fcell.2021.736267)
Supplement: Supplementary file 1 [file DataSheet1.PDF]

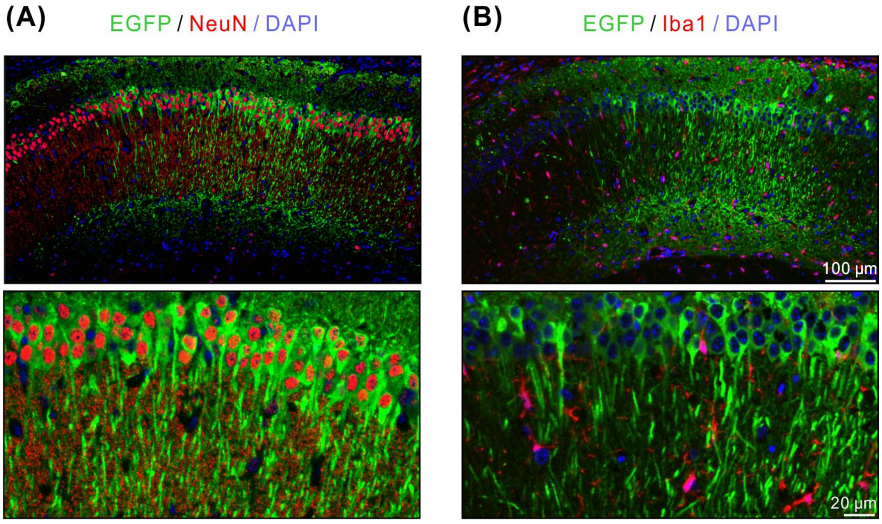

Supplementary Figure 1. Exogenous PINK1 is over-expressed in the neurons.
The EGFP- fused virus constructs pAAV-SYN-PINK1-EGFP-3×FLAG-WPRE was infused into the hippocampal CA1 subset of 2-month-old C57 mice for 1 month, and then expression of exogenous PINK1, Neuron-specific nuclei protein (NeuN), and Iba1 (a microglia marker) were shown by immunofluorescence.

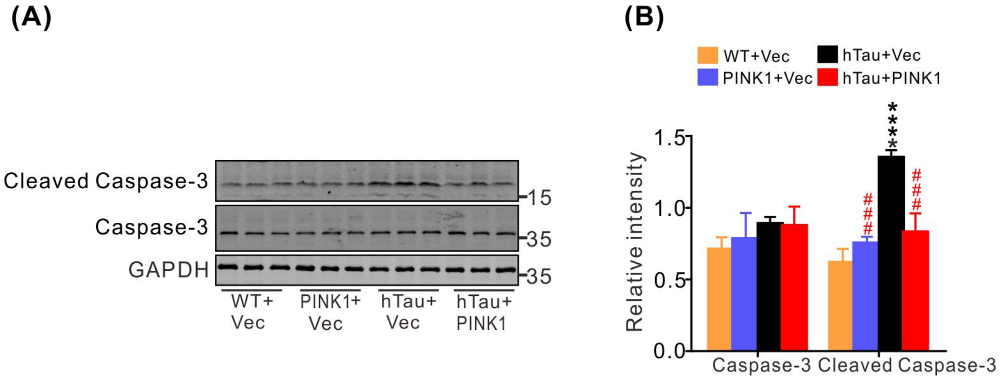

Supplementary Figure 2. PINK1 attenuates the increased levels of cleaved caspase-3 in hTau mice
(A) PINK1 decreased the levels of cleaved caspase-3 in the hippocampal CA1 region of hTau mice detected by Western blot. **(B)** Quantitative analyses of the levels of caspase-3 and cleaved caspase-3. One-way ANOVA followed by Tukey multiple-comparisons tests. ****, p <0.0001 *vs.* WT + Vec; ###, *p*<0.001 *vs.* hTau + Vec. All data were presented as mean ± SD. n = 3 mice for each group.

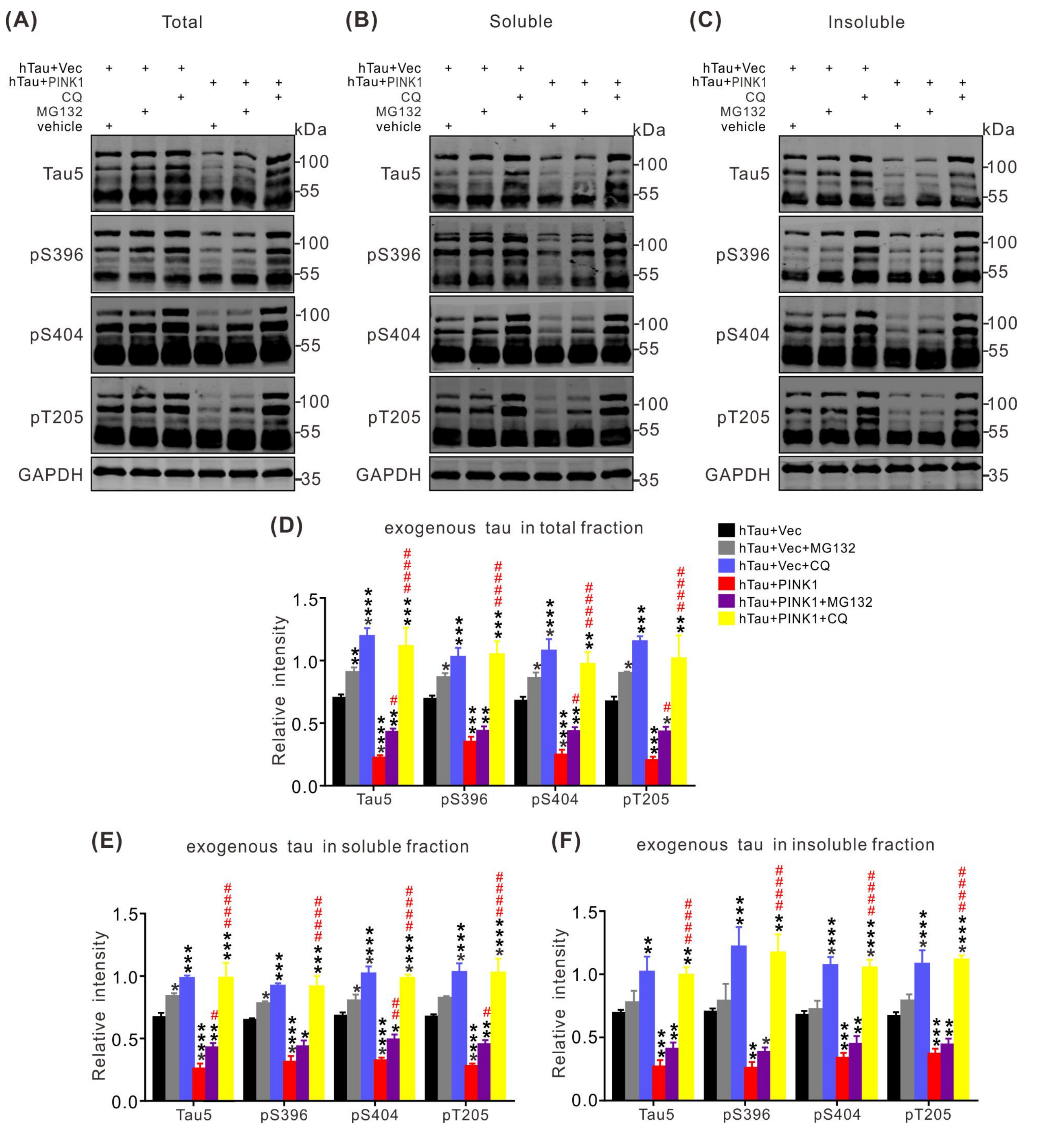

Supplementary Figure 3. PINK1 reduces the levels of hTau while CQ reverses this effect

(A, B, C) In the total **(A)**, soluble **(B)** and insoluble **(C)** fraction of mice hippocampal CA1 area, PINK1 attenuated exogenous tau (~106kDa, human tau: both total and phosphorylated tau) levels, while CQ treatment reversed this reduction shown by Western blot.

(D, E, F) Quantitative analyses of the levels of hTau proteins. One-way ANOVA followed by Tukey multiple-comparisons tests. *, $p<0.05$, **, $p<0.01$, ***, $p<0.001$, ****, $p<0.0001$ vs. hTau + Vec; #, $p<0.05$, ##, $p<0.01$, ###, $p<0.001$, ####, $p<0.0001$ vs. hTau + PINK1. All data were presented as mean ± SD. n = 3 mice for each group.

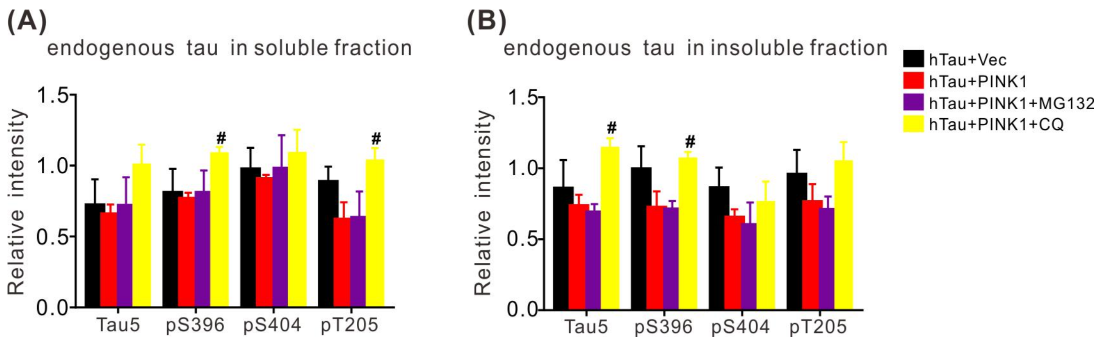

Supplementary Figure 4. CQ treatment increases endogenous mouse tau.

CQ treatment increased endogenous (mouse) tau (both total and phosphorylated tau) in the soluble **(A)** and insoluble fraction **(B)** of mice hippocampal CA1 region, the blots were shown in Figure 5E, F (endogenous tau, ~55 kDa). One-way ANOVA followed by Tukey multiple-comparisons tests. #, $p<0.05$ *vs.* hTau + PINK1. All data were presented as mean ± SD. n = 3 mice for each group.

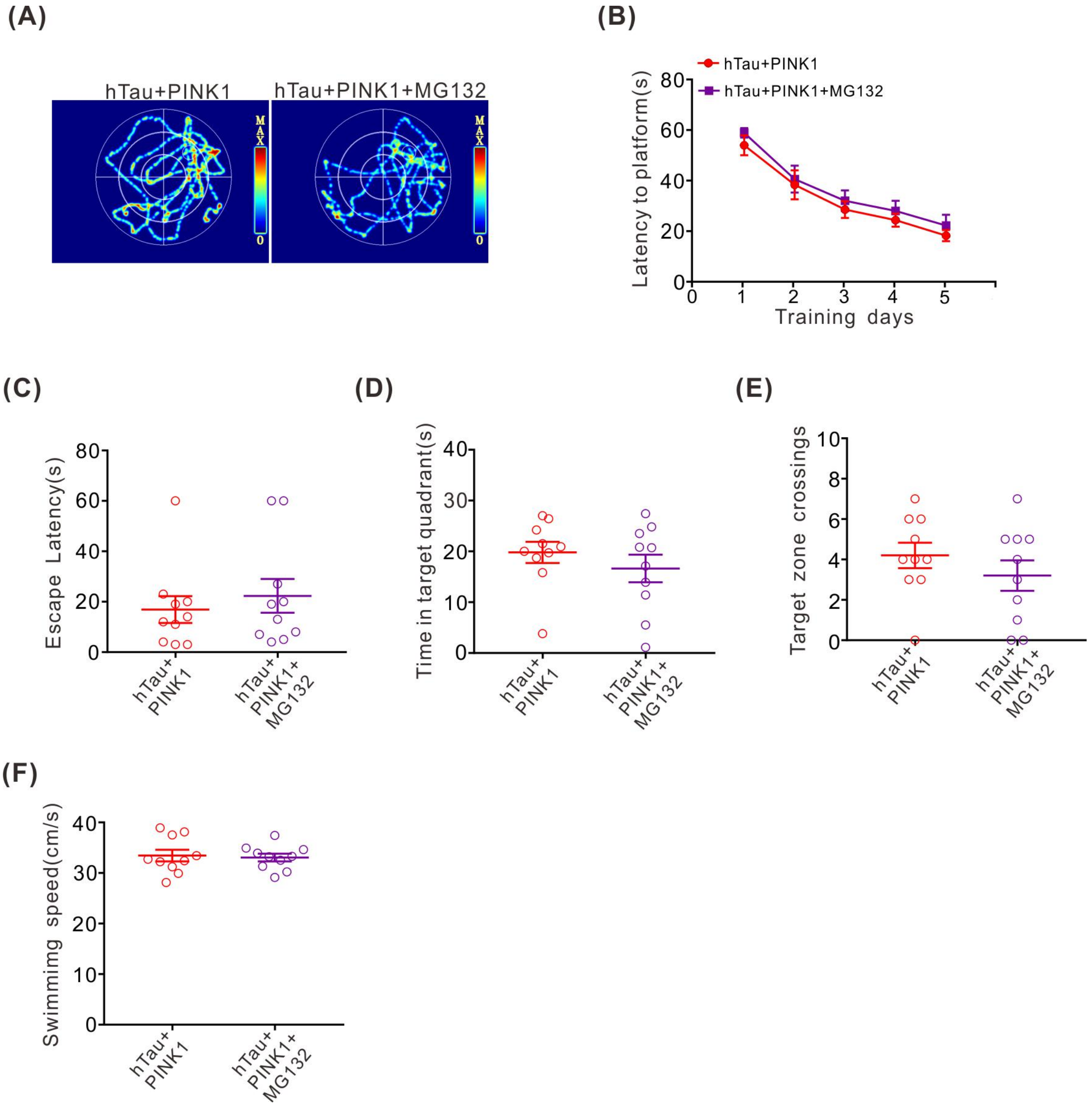

Supplementary Figure 5. MG132 has no effects in the cognitive ability of hTau and PINK1 overexpressing mice.

MG132 treatment did not induce significant cognitive changes in hTau and PINK1 overexpressing mice, shown by the MWM test. **(A)** Representative swimming path of mice in each group during the MWM probe test; **(B)** Latency to reach hidden platform during the training phase. Two-way repeated-measures ANOVA followed by Tukey multiple-comparisons tests; **(C)** Escape latency, **(D)** residence time in the target quadrant, **(E)** platform zone crossing times and **(F)** swimming speed during the test phase of the MWM test. Unpaired t-tests. All data were presented as mean ± SEM. n = 10 mice for each group.

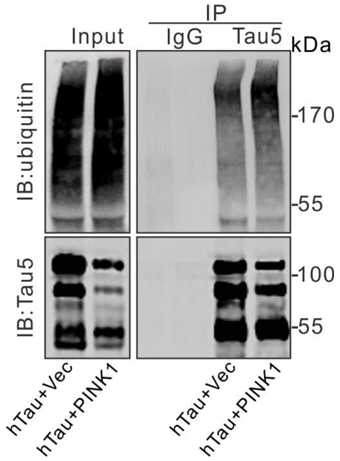

Supplementary Figure 6. PINK1 increases the ubiquitination of tau.
Overexpression of PINK1 increased the ubiquitination of tau detected by Co-IP and Western blot. n = 3 mice for each group.
